# Quantifiable and reproducible phenotypic assessment of a constitutive knockout mouse model for congenital nephrotic syndrome of the Finnish type

**DOI:** 10.1038/s41598-024-64883-y

**Published:** 2024-07-10

**Authors:** Katharina Lemberg, Nils D. Mertens, Kirollos Yousef, Ronen Schneider, Lea M. Merz, Bshara Mansour, Daanya Salmanullah, Caroline M. Kolvenbach, Ken Saida, Seyoung Yu, Selina Hölzel, Andrew Steinsapir, Kevin A. Goncalves, Camille Nicolas Frank, Gijs A. C. Franken, Shirlee Shril, Florian Buerger, Friedhelm Hildebrandt

**Affiliations:** 1 Division of Nephrology, Department of Pediatrics, Boston Children’s Hospital, Harvard Medical School, Boston, Massachusetts USA; 2 Medical Faculty, Institute of Anatomy, University of Bonn, Bonn, Germany; 3 Deerfield Discovery and Development, Deerfield Management Company, L.P. (Series C), New York, NY USA; 4 Department of Pediatrics, University Hospital Leipzig, Leipzig, Germany; 5 University Children’s Hospital, University Medical Center Hamburg-Eppendorf, Hamburg, Germany

**Keywords:** Cell biology, Genetics, Molecular medicine, Nephrology, Pathogenesis

## Abstract

Steroid-resistant nephrotic syndrome (SRNS) is the second most frequent cause of childhood chronic kidney disease. Congenital nephrotic syndrome of the Finnish type (CNF) (MIM# 256300) is caused by biallelic variants in the gene *NPHS1*, encoding nephrin, an integral component of the kidney filtration barrier. No causal treatments exist, and children inevitably require kidney replacement therapy. In preparation for gene replacement therapy (GRT) in CNF, we established a quantifiable and reproducible phenotypic assessment of the nephrin-deficient CNF mouse model: 129/Sv-*Nphs1*^*tm1Rkl*^*/J*. We assessed the phenotypic spectrum of homozygous mice (*Nphs1*^*tm1Rkl*^*/Nphs1*^*tm1Rkl*^) compared to heterozygous controls (*Nphs1*^*tm1Rkl*^*/Nphs1*^*WT*^) by the following parameters: 1. cohort survival, 2. podocyte foot process (FP) density per glomerular basement membrane (GBM) using transmission electron microscopy, 3. tubular microcysts in brightfield microscopy, and 4. urinary albumin/creatinine ratios. *Nphs1*^*tm1Rkl*^*/Nphs1*^*tm1Rkl*^ mice exhibited: 1. perinatal lethality with median survival of 1 day, 2. FP effacement with median FP density of 1.00 FP/µm GBM (2.12 FP/µm in controls), 3. tubular dilation with 65 microcysts per section (6.5 in controls), and 4. increased albumin/creatinine ratio of 238 g/g (4.1 g/g in controls). We here established four quantifiable phenotyping features of a CNF mouse model to facilitate future GRT studies by enabling sensitive detection of phenotypic improvements.

## Introduction

Steroid-resistant nephrotic syndrome (SRNS) is the second most frequent cause of chronic kidney disease in the first three decades of life^1,2^. In approximately 25% of SRNS cases, a pathogenic variant can be detected in one of ~ 70 genes known to cause monogenic forms of SRNS^3,4^. The most severe and second most frequent cause of monogenic SRNS (4.3% of cases) are biallelic pathogenic variants in *NPHS1*^4^. The *NPHS1* gene (NM_004646) is predominantly expressed by glomerular podocytes of the kidney and encodes nephrin, a 135 kDa protein, forming an integral structural part of the glomerular slit membrane. Individuals with biallelic pathogenic variants in *NPHS1* develop congenital nephrotic syndrome of the Finnish type (CNF), also referred to as congenital nephrotic syndrome type 1^3^. CNF manifests with severe nephrotic syndrome in utero (MIM# 256300). Current treatment strategies focus on maintaining intravascular euvolemia and optimizing nutrition while preventing complications such as hemodynamic instability, infections, thromboses, impaired growth, and slowing progression of kidney failure. Most affected children develop end-stage kidney disease requiring kidney replacement therapy within the first 2–3 years of life^5^. Bilateral nephrectomies are recommended for patients with severe complications or persistent nephrotic syndrome to prevent excessive wasting of crucial serum proteins through urinary loss^6^. To date, no causal treatment strategy has been established. During the past years, gene replacement therapy (GRT) has become more feasible and continues to enter clinical trials for a wide variety of diseases. Specifically, two important studies have additionally demonstrated first therapeutic effects in monogenic SRNS by means of AAV-mediated GRT. Ding et al. have achieved delivery of *Nphs2* cDNA to podocytes of Nphs2-deficient mice, positively affecting disease course^7^. Zhao et al. demonstrated in a pioneering study that, although a more systemic disease, monogenic SRNS due to S1P lyase insufficiency syndrome can be effectively treated through gene transfer in mice^8^. For CNF, there are multiple mouse models available, for instance the following: 1. constitutive nephrin-deficient 129/Sv-*Nphs1*^*tm1Rkl*^*/J* mice^9^, 2. *129Sv.Nphs1*^*fl*^ mice with *loxP* sites flanking exon 5 of the *Nphs1* gene (*129Sv.Nphs1*^*1tm1.1Pgarg*^*/J*) allowing for conditional expression of truncated nephrin protein lacking the transmembrane and intracellular domains^10^, and 3. *C57BL/6J.Nphs*^*fl*^ mice with *loxP* sites flanking exons 1B-5 of the *Nphs1* gene (*B6(DBA)-Nphs1*^*tm1Afrn*^*/J*) to be used for conditional nephrin deletion^11^. We acquired and investigated 129/Sv-*Nphs1*^*tm1Rkl*^*/J* knockout mice, as their allele genetically resembles the most common pathogenic *NPHS1* variant in humans NM_004646.4:c.121_122del (p.Leu41fs), termed Fin-major, which causes a frameshift and premature stop codon in exon 2 in *NPHS1*^5^*.* As a prerequisite for developing gene replacement approaches for CNF as a causal therapeutic strategy, we here established quantifiable and reproducible phenotyping of the constitutive nephrin-deficient CNF mouse model 129/Sv-*Nphs1*^*tm1Rkl*^*/J*^9^*.*

## Results

To enable future gene replacement therapy (GRT) approaches towards CNF, we here attempted to establish robust quantifiable and reproducible phenotyping parameters for the constitutive nephrin-deficient CNF mouse model: 129/Sv-*Nphs1*^*tm1Rkl*^*/J*^9^. Homozygous mice (*Nphs1*^*tm1Rkl*^/*Nphs1*^*tm1Rkl*^) will be referred to as *Nphs1*^*−/−*^, heterozygous controls (*Nphs1*^*tm1Rkl*^*/Nphs1*^*WT*^) as *Nphs1*^+*/−*^ mice, and wildtype mice as *Nphs1*^+*/*+^ mice. In total, 302 mice were born in 84 litters from 34 breeding pairs in non-Mendelian ratios (77 *Nphs1*^+*/*+^, 25.5%; 178 *Nphs1*^+*/−*^, 59%; 47 *Nphs1*^*−/−*^, 15.5%) which was consistent with known intrauterine disease onset and thus potential perinatal lethality^9^. First, complete absence of nephrin in *Nphs1*^*−/−*^ mice was confirmed by highly sensitive immunofluorescence imaging using a well-established anti-nephrin antibody and control co-staining of podocyte nuclei for WT1 (Supplementary Fig. 1).

The four phenotypic parameters that we decided to assess include the following: 1. Kaplan–Meier survival analysis, 2. evaluation of podocyte foot process density as the number of podocyte foot processes per µm of GBM, 3. evaluation of the number of kidney tubular microcysts, and 4. quantification of urinary albumin/creatinine ratios.

### *Nphs1*^*−/−*^ mice show a median survival of 1 day

**Figure 1:**
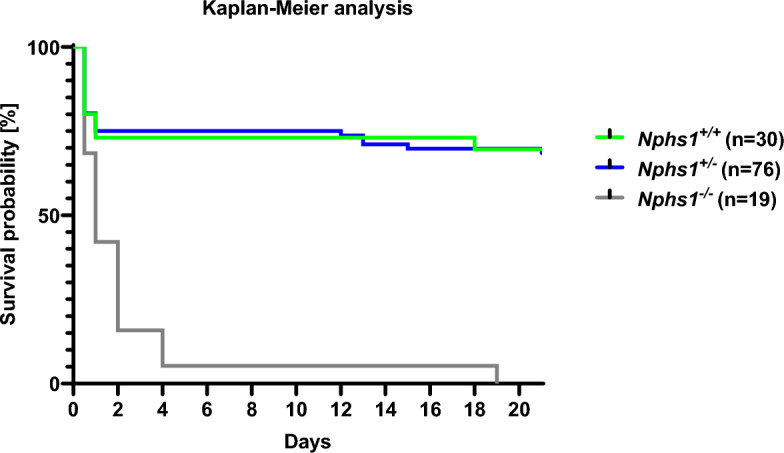
Kaplan–Meier survival analysis: *Nphs1*^*−/−*^ mice have a median survival probability of one day. 11 *Nphs1*^+*/−*^ breeding pairs gave birth to a total of 38 litters with 125 newborn (30 WT, 76 *Nphs1*^+*/−*^, 19 *Nphs1*^*−/−*^). 95% of *Nphs1*^−/−^ mice were found dead at P4 (grey line). One *Nphs1*^−/−^ mouse survived for a maximum of 19 days. 25% *Nphs1*^+*/*+^ (green line) and 25% *Nphs1*^+*/−*^ (blue line) control animals were found dead within the first day after birth. 95% of the remaining controls lived until weaning age at 21 days. Animals that were found dead in the morning after delivery were considered dead at P0.5. Log-rank (Mantel-Cox) test for *Nphs1*^+*/*+^ vs. *Nphs1*^+*/−*^ P = 0.9, for *Nphs1*^+*/*+^ vs. *Nphs1*^*−/−*^ P < 0.01, for *Nphs1*^+*/−*^ vs. *Nphs1*^*−/−*^ P < 0.01.

Kaplan–Meier survival analysis included data from 125 mice born to 11 *Nphs1*^+*/−*^ breeding pairs in 38 litters (Fig. 1). *Nphs1*^*−/−*^ mice had a median survival probability of one day. 90% of *Nphs1*^−/−^ mice died within the first 4 days of life. One *Nphs1*^−/−^ mouse survived to a maximum of 19 days. 25% of *Nphs1*^+*/*+^ and *Nphs1*^+*/−*^ control animals died within the first day after birth while 95% of the remaining control mice lived until weaning at the age of 21 days, the last day of surveillance (Fig. 1). The survival between *Nphs1*^+*/*+^ and *Nphs1*^+*/−*^ did not differ significantly (Fig. 1).

### The podocyte foot process density of newborn *Nphs1*^*−/−*^ mice is dramatically reduced to 50%

**Figure 2:**
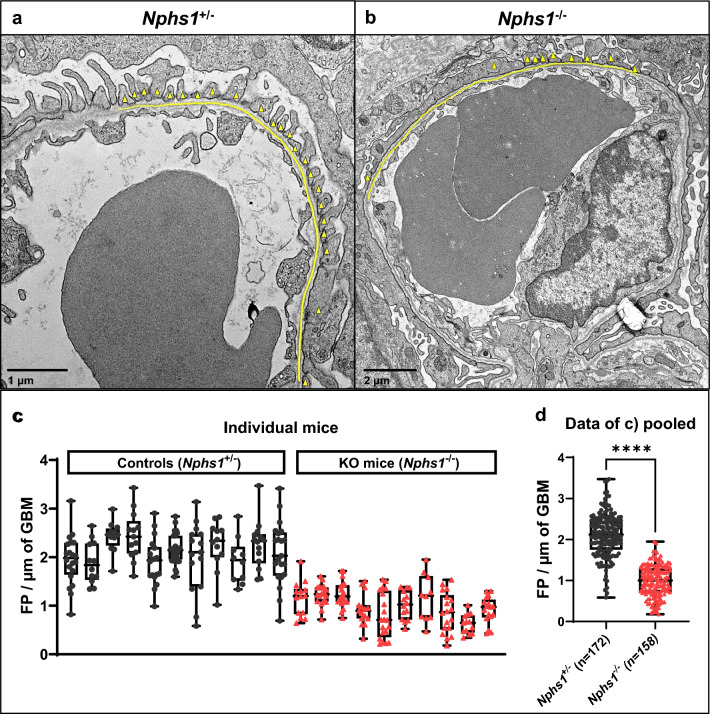
Podocyte foot process density is significantly reduced in homozygous *Nphs1*^*−/−*^ mice compared to control animals as assessed by transmission electron microscopy imaging. Representative TEM images of heterozygous *Nphs1*^+*/−*^ control (**a**) and homozygous *Nphs1*^*−/−*^ mice (**b**). The evaluation criteria to assess the number of FP/µm of GBM were: 1. symmetric *Laminae rara interna, densa, and externa* of the GBM, and 2. rhythmicity of adjacent endothelial fenestrae. The extent to which these criteria were met along a continuous stretch of GBM was denoted by drawing a yellow line in *ImageJ*. (**c,d**) Quantitation of number of foot processes per µm of linear GBM was performed as described in (**a,b**). *Nphs1*^+/−^ control mice (n = 11) had a mean of 2.15 FP/µm of GBM. In comparison, *Nphs1*^*−*/−^ mice (n = 10) had a mean of 0.98 FP/µm of GBM. X-axis positions relate to individual mice. (**c**) Each dot represents a single evaluated TEM image belonging to the same mouse. (**d**) Pooled numbers of podocyte foot processes per GBM (from **c**). Box plots denote median, quartiles and range. P value for two-tailed Mann–Whitney test between groups: P < 0.0001. Yellow triangles: individual podocyte foot processes. Yellow line: GBM. Magnification: (**a**) × 4800, (**b**) × 2900.

Due to early lethality of *Nphs1*^*−/−*^ mice, we performed all further phenotyping at the ages P0-P1. Reproducible quantification of podocyte foot processes (FP) per µm of glomerular basement membrane (FP/µm of GBM) was achieved by counting FP per length of perpendicularly sectioned GBM (Fig. 2a,b). Predefined criteria for identifying perpendicularly sectioned stretches of GBM included symmetry of the GBM *Laminae rara interna, densa,* and *externa* and rhythmicity of adjacent endothelial fenestrae. More than 10 images of at least three glomeruli were evaluated for each mouse (Fig. 2c,d). *Nphs1*^+*/−*^ control mice (n = 11) showed a median density of 2.12 FP/µm of GBM while *Nphs1*^*−/−*^ mice (n = 10) had a significantly lower median density of 1.00 FP/µm of GBM (P < 0.0001).

### *Nphs1*^*−/−*^ mice exhibit a high number of kidney tubular microcysts

**Figure 3:**
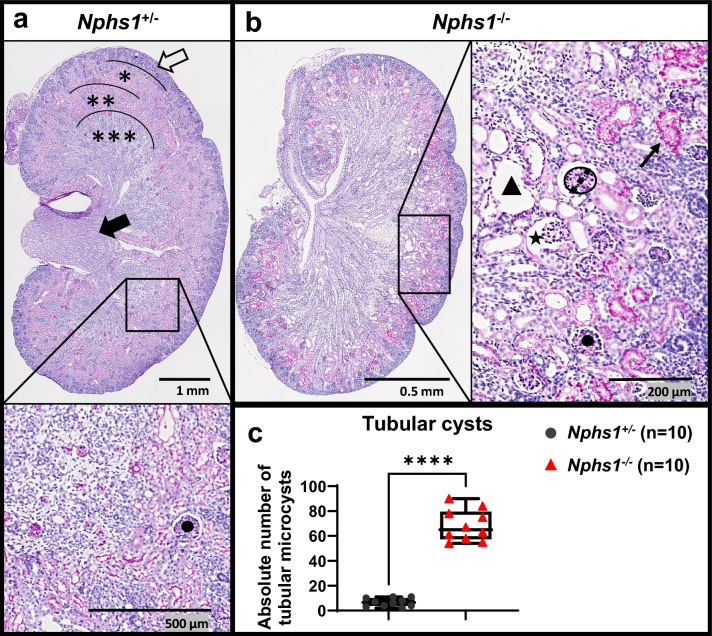
Quantification of kidney tubular microcyst upon light microscopy. PAS-stained coronal equatorial kidney sections of 10 *Nphs1*^+*/−*^ control mice (**a**) and 10 *Nphs1*^*−/−*^ mice (**b**) were imaged and evaluated according to the following standardized steps: 1. identify the cortical nephrogenic zone (outer black delimiter at white filled arrow in **a**), cortex (*), outer medulla (**), inner medulla (***), and kidney papilla (filled black arrow in **a**), 2. highlight all mature glomeruli (e.g., black dot in **a,b**), 3. determine the “standard critical surface area” (SCSA) by measuring the vertical (central) diameter of an equatorially cut mature glomerulus featuring a recognizable capillary tuft or urinary pole (dashed line crossing a mature glomerulus encircled by a black line in **b**), and 4. count the number of proximal tubules (black filled triangle in **b**) with a diameter exceeding half the SCSA diameter. *Nphs1*^*−/−*^ often showed PAS-positive material in the cytoplasm of proximal convoluted tubules (black arrow in **b**). We counted glomerular cysts (e.g., black filled star in **b**) separately (see Suppl. Fig. 2). (**c**) *Nphs1*^*−/−*^ mice had a median of 65 kidney tubular microcysts (range 54 to 90) per section, whereas *Nphs1*^+*/−*^ control mice had a median of 6.5 microcysts (range 2 to 11) per kidney section (difference between means 61.9 ± 4.1). Two-tailed Mann–Whitney test between groups: P < 0.0001.

Most prominently, *Nphs1*^*−/−*^ mice exhibited kidney tubular microcysts. To quantify this phenotype, we counted kidney tubular microcysts as described in Fig. 3a,b (and in the “Methods” section). *Nphs1*^*−/−*^ mice exhibited a median of 65 kidney tubular microcysts per section (range 54–90), whereas the median in *Nphs1*^+*/−*^ control mice was 6.5 microcysts per kidney section (range 2–11) (P < 0.0001) (Fig. 3c). Moreover, *Nphs1*^*−/−*^ mice had a significantly increased number of glomerular cysts (dilation of the Bowman’s space) with a median of 12 glomerular microcyst per section (range 4–21) *vs.* a median of 2 in *Nphs1*^+*/−*^ control mice (range 0–2) (P < 0.0001) (Supplementary Fig. 2a). The number of glomerular microcysts was less consistent across *Nphs1*^*−/−*^ mice than the evaluated kidney tubular microcysts. The overall number of mature glomeruli was similar in *Nphs1*^*−/−*^ mice and controls (Supplementary Fig. 2b).

### Urinary albumin/creatinine ratio

**Figure 4:**
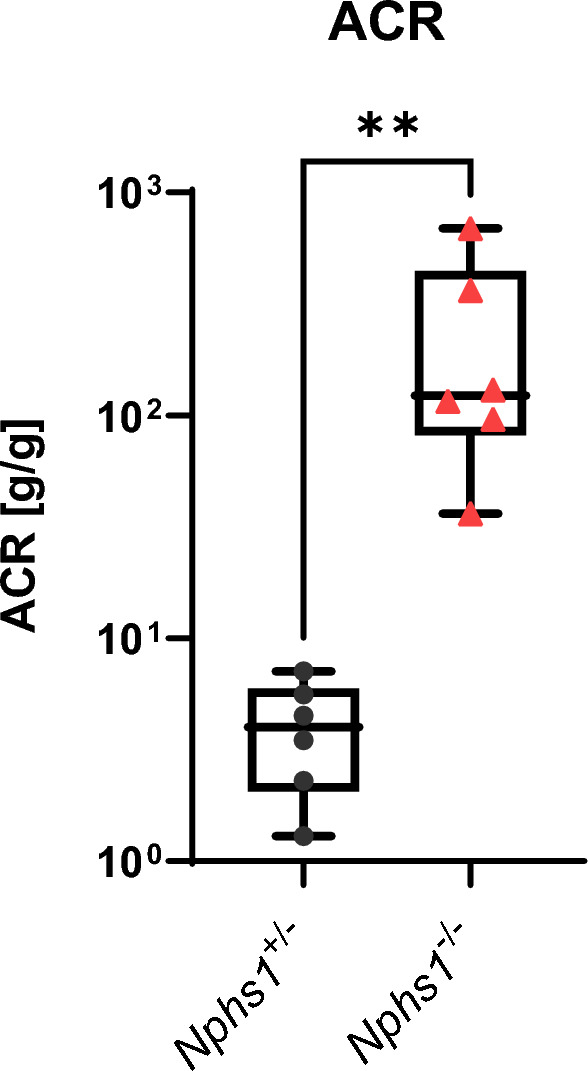
*Nphs1*^*−/−*^ mice display severe proteinuria upon birth. We performed urine albumin and urine creatinine measurements in 6 *Nphs1*^+*/−*^ control and 6 *Nphs1*^*−/−*^ mice at P0-P1 to calculate urine albumin/creatinine ratios (uACR). Two-tailed Mann–Whitney test for uACR P < 0.01.

Urinary albumin/creatinine ratios were determined as single time-point comparisons after collecting urine samples of 6 *Nphs1*^+*/−*^ and 6 *Nphs1*^*−/−*^ mice by bladder puncture at P0-P1 during the dissection necessary for ultrastructural and histologic analyses. Due to the extremely low survival rate of *Nphs1*^*−/−*^ mice, a dynamic multi time-point measurement was not possible. Urine analysis demonstrated massive proteinuria in *Nphs1*^*−/−*^ mice with a median albumin/creatinine ratio of 238 g/g in *Nphs1*^*−/−*^* vs.* 4.1 g/g in *Nphs1*^+*/−*^ controls (P < 0.01) (Fig. 4).

## Discussion

We, here, developed phenotypic parameters for CNF in the published nephrin-deficient CNF mouse model 129/Sv-*Nphs1*^*tm1Rkl*^*/J* by performing 1. Kaplan–Meier survival analysis, 2. evaluation of podocyte foot process density as the number of podocyte foot processes per µm of GBM, 3. evaluation of the number of kidney tubular microcysts, and 4. measurements of urinary albumin/creatinine ratios. All four phenotyping parameters were quantifiable, reproducible, and highly distinctive between *Nphs1*^*−/−*^ mice and controls. These phenotypic parameters will facilitate evaluation of therapeutic effects conveyed by future GRT studies.

Of note, apart from survival analyses, we chose to only compare knockout animals (*Nphs1*^*−/−*^) to heterozygous control animals (*Nphs1*^+*/−*^) to replicate conditions of a recessive disease. Although heterozygous allele carrying parents or siblings of patients affected by CNF are generally regarded as healthy, evidence for heterozygous *NPHS1* variants in humans being associated with nephrotic syndrome is emerging^12,13^. Therefore, we exclusively evaluated parameters in comparison to heterozygous animals to respect these recent observations and to avoid general off-target effects of transgenic mouse alleles.

### Kaplan–Meier survival analysis

Hamano et al. report that constitutively nephrin-deficient mice die within 2 days after birth^9^. Here, we performed Kaplan–Meier survival analysis to assess the reproducibility of this parameter as the most clinically relevant end-point parameter. We found that over half of *Nphs1*^*−/−*^ mice died within the first day, and 95% of *Nphs1*^*−/−*^ mice died within four days after birth. Moreover, we observed perinatal lethality in about 25% of *Nphs1*^+*/−*^ and *Nphs1*^+*/*+^ littermate controls. In contrast to the rapid postnatal decline in survival probability of *Nphs1*^*−/−*^ mice, 95% of the remaining *Nphs1*^+*/−*^ and *Nphs1*^+*/*+^ littermate controls at day 2 lived beyond the end of the 21-day total surveillance period. Considering the absence of perinatal lethality in simultaneously performed maintenance breeding of *Nphs1*^+*/*+^ and *Nphs1*^+*/−*^ animals, our data suggests a deleterious impact of diseased *Nphs1*^*−/−*^ mice on their *Nphs1*^+*/−*^ and *Nphs1*^+*/*+^ littermates. This effect could be conveyed by in utero effects that align with massive prenatal proteinuria observed in CNF patients^5^. Alternatively, morbidity and early lethality of diseased *Nphs1*^*−/−*^ mice is likely to cause stress leading to poor nursing and potentially the mother abandoning or even cannibalizing their young regardless of individual genotype. The latter could be further assessed by fostering newborn litters onto nursing surrogate mothers.

Our results from the Kaplan–Meier survival analysis in the here studied mouse model suggest a prenatal therapeutic window for CNF gene replacement and will therefore require fetal gene delivery which could be achieved through tail vein injection to pregnant dams at around E12 as has been demonstrated before^14^. CNF mouse models relying on Cre recombinase-driven *Nphs1* knockout may have a wider therapeutic window due to a promoter-dependent but delayed Cre activation^10,11^. However, unreliable Cre activity in those mouse models further complicates interpretation of subtle therapeutic effects due to higher phenotypic variability. The here studied constitutive *Nphs1* knockout mouse model has the advantage of closely recapitulating the severe early onset of human CNF. As it is the only *Nphs1* knockout mouse model that does not express residual nephrin protein, it also allows for reliable testing of *Nphs1* transgene expression and nephrin localization without the necessity of artificial molecular tags.

### Ultrastructural phenotype quantitation

The loss of functional nephrin in humans is known to cause a variety of ultrastructural changes, apparent upon inspection of kidney sections with electron microscopy. The most prominent features include a fusion of podocyte foot processes, reduced numbers of slit pores, and ultimately the absence of the entire slit diaphragm^5^. We sought to identify the most robust and reproducible quantification method for ultrastructural changes in nephrin-deficient mice, similar to a method described in human fetuses with CNF^15^. Our data demonstrate that podocyte foot process density is a highly distinctive and reproducible feature between *Nphs1*^*−/−*^ mice and *Nphs1*^+*/−*^ controls. Control animals exhibit a foot process density of 2.12 FP/µm of GBM (range 0.69 to 2.54 FP/µm of GBM), which is about twice as high as in *Nphs1*^*−/−*^ mice with 1.00 FP/µm of GBM (range 0.34 to 1.3 FP/µm of GBM) (Fig. 2c,d). Of note, the thickness of glomerular basement membranes did not differ between groups (Supplementary Fig. 2c).

Foot process effacement represents the first morphologically resolvable step in the pathogenesis of NS and is closely linked to the molecular cause of CNF, the loss of functional nephrin molecules that promote cell–cell adhesions through homophilic interactions^16^. Therefore, ultrastructural phenotype quantification may offer the highest specificity among the here established phenotyping methods and could be used to assess therapeutic effects on a single glomerular or even molecular level. The latter could be achieved by combining FP density assessment with immunogold labeling of nephrin protein during transmission electron microscopy.

### Histopathological phenotype quantitation

CNF was first described by Ahvenainen et al. in 1956^17^. In the following years, multiple groups studied the histopathological phenotype of CNF in human fetuses and infants^18–24^. The most consistently described phenotypes included: 1. increased number and size of mature glomeruli^18,19^, 2. mesangial hypercellularity^21,22^, 3. glomerular sclerosis along with dilation of glomerular urinary space, 4. kidney proximal tubular microcysts within the kidney cortex^21^, and 5. tubular atrophy^21^. Additionally, proximal tubules of patients with CNF often contain eosinophilic granular debris, which is described as proteinaceous casts and deemed pathognomonic for CNF^24^.

Newborn mouse kidneys at P0-P12 resemble human fetal kidneys. Therefore, we focused the phenotypic light microscopy evaluation on counting the number of kidney tubular microcysts, which are described to manifest already at gestational ages 16–24 weeks in human CNF cases. The term tubular microcyst is here used in line with the literature and describes the dilation of proximal kidney tubules^21,24^. Microcyst development is proposed to precede tubular atrophy, interstitial fibrosis, and mesangial hypercellularity^18^. We quantified the relative number of kidney tubular microcysts by establishing a reproducible phenotyping method and found consistent numbers of tubular microcysts within *Nphs1*^*−/−*^ mice and *Nphs1*^+*/−*^ controls, respectively (Fig. 3c). The numbers were highly distinctive between both groups. We also observed a prominent accumulation of PAS-positive material in the cytoplasm of proximal convoluted tubular cells in *Nphs1*^*−/−*^ mice, along with a varying fraction of proximal tubular lumens to contain proteinaceous casts. In addition to kidney tubular microcysts, *Nphs1*^*−/−*^ mice had significantly increased numbers of glomerular cysts (Supplementary Fig. 2a). We observed multiple glomerular microcysts that extended into the urinary pole of the Bowman’s space and therefore speculate that the glomerular microcysts are an extension of the kidney tubular microcysts or vice versa.

In summary, Our assessments, including ultrastructural, histopathological, and urine analysis, demonstrate that the studied mouse model phenotypically closely recapitulates human CNF. We here established four robust, quantifiable, and reproducible phenotyping methods for the *Nphs1*^*[tm1Rkl/J]*^ mouse model as a prerequisite to studying the potential effects of future gene replacement projects in CNF.

## Methods

### Mouse breeding and maintenance

The experimental animal protocols were reviewed and approved by the Institutional Animal Care and Use Committee at the Boston Children’s Hospital (00001522). Animal research presented throughout the current study was completed and reported in accordance with ARRIVE guidelines. Mice were handled according to the Guidelines for the Care and Use of Laboratory Animals and housed under pathogen-free conditions with a light period from 7:00 AM to 7:00 PM and ad libitum access to water and rodent chow. The transgenic 129/Sv-*Nphs1*^*tm1Rkl*^*/J* mouse strain was generated by Hamano et al. in 2002 by inserting a transgene containing the mouse *Nphs1* promoter and the beginning of *Nphs1* exon 1 in frame with a GFP-pA/PGK-Neo-pA cassette into a unique *SacII* site for negative selection in embryonic stem (ES) cells. Mice were bred on a pure 129/Sv mouse background^9^. We rederived the mouse model through The Jackson Laboratory (JAX) (Strain #005692). Genotyping was performed by multiplex PCR using the following primers: 1. a sense primer (5’-CAC AAG GGA AGA TGG AGG AGT TG-3') for the *Nphs1* gene, 2. an antisense primer (5’-TCC ACT CAC CTG TGG TCA GCA TTC-3') for the *Nphs1* gene, and 3. an antisense primer (5’-AGT CGT GCT GCT TCA TGT GGT C-3') for the GFP sequence.

### Kaplan–Meier survival analysis

We designated breeding pairs for Kaplan–Meier analysis a priori. Newborn mice were genotyped on the day of birth and then monitored twice daily following the experimental animal protocol with minimal handling.

### Ultrastructural analysis

Sample preparation: Newborn mice at P0-P1 were sacrificed through decapitation. Kidneys were resected, and poles were fragmented into approximately 1 mm^3^ large tissue samples. Samples were collected in 2.5% glutaraldehyde, 1.25% paraformaldehyde (PFA), and 0.03% picric acid in 0.1 M sodium cacodylate buffer (pH 7.4) and incubated overnight at 4 °C. The samples were then washed with 0.1 M phosphate buffer, post-fixed with 1% OsO4 dissolved in 0.1 M phosphate-buffered saline (PBS) for 2 h, dehydrated in an ascending gradual series (50‒100%) of ethanol, and infiltrated with propylene oxide. Samples were embedded using the Poly/Bed 812 kit (Polysciences) according to the manufacturer’s instructions. After pure fresh resin embedding and polymerization in a 65 °C oven (TD-700, DOSAKA, Japan) for 24 h, approximately 200–250 nm thick sections were stained with toluidine blue for light microscopy. 70 nm thick sections were double stained with 6% uranyl acetate (Electron Microscopy Sciences, 22400) for 20 min and lead citrate (Thermo Fisher Scientific) for 10 min for contrast enhancement. The samples were sectioned using Reichert Ultracut S/Leica EM UC-7 (Leica) with a diamond knife (Diatome) and transferred onto copper and nickel grids. The sections were evaluated by transmission electron microscopy (Tecnai G^2^ Spirit BioTWIN) at an acceleration voltage of 80 kV.

Analysis procedure: Blinded image acquisition was performed by independent evaluators. For each animal of the 10 *Nphs1*^*−/−*^ knockout and 10 *Nphs1*^+*/−*^ control mice at days P0-P1, 15–20 images of > 3 glomeruli showing areas with symmetric GBM laminae were acquired. *ImageJ* was used to assess the podocyte foot process density, which we quantified according to the following standardized protocol: Stretches of the glomerular basement membrane (GBM) were identified and included in the analysis if the GBM *laminae* (*rara interna*, *rara densa*, and *rara externa*) were symmetric and the adjacent endothelial fenestrae rhythmic, indicating a perpendicular section of the GBM. A line was drawn along the GBM using *ImageJ* to measure the length in μm and to denote the stretch of the GBM where these criteria were met. The number of foot processes was counted along this denoted stretch of GBM (Fig. 2a,b). Finally, the ratio of foot processes per length of the denoted stretch of GBM was calculated.

### Histopathological analysis

Sample preparation: Mice were sacrificed at P0 through decapitation. After dissection, whole kidneys were fixed in 4% PFA overnight, washed with PBS, and embedded in Paraffin following standard dehydration steps. Paraffin-embedded kidneys were sectioned into five μm thick sections and stained with periodic acid-Schiff (PAS), Masson's trichrome (MT), and Hematoxylin–Eosin (HE), following standard protocols.

Analysis procedure: Blinded image acquisition was performed by independent evaluators. PAS-stained coronal equatorial kidney sections of 10 *Nphs1*^*tm1Rkl*^/*Nphs1*^*tm1Rkl*^ knockout and 10 *Nphs1*^*tm1Rkl*^*/Nphs1*^*WT*^ control mice were imaged using a Nikon Eclipse Ni microscope and evaluated according to the following standardized steps: 1. identify the different kidney zones from outside to inside: cortical nephrogenic zone, cortex, outer medulla, inner medulla, and kidney papilla (legend, Fig. 3a), 2. highlight all mature glomeruli (mature glomeruli were defined by their location and appearance, glomeruli within the nephrogenic zone were excluded from further analysis) (Fig. 3b), 3. determine the “standard critical surface area” (SCSA) by measuring the vertical (central) diameter of an equatorially sectioned mature glomerulus featuring a recognizable capillary tuft or urinary pole (Fig. 3b), and 4. count the number of proximal tubules with a diameter exceeding half the SCSA diameter.

### Immunofluorescence studies

After dissection at P0–P1, whole kidneys of *Nphs1*^-/-^ knockout and 10 *Nphs1*^+/-^ control mice were embedded in OCT and snap frozen. After sectioning, tissue was fixed in 4% paraformaldehyde for 3 minutes and permeabilized using 0.1% Triton-X-100. After blocking, primary antibodies were applied and the sections incubated overnight at 4°C, followed by incubation in secondary antibodies for 90 min at room temperature. Subsequently, sections were incubated with DAPI (D1306, Invitrogen) for 5 minutes and mounted using ProLong Gold Antifade Mountant (Invitrogen). Confocal microscopy imaging was performed using the Leica SP5X system with an upright DM6000 microscope, and images were processed with the Leica AF software suite.

### Antibodies

The following antibodies were used: anti-nephrin (GP-N2, Progen), anti-WT1 (83535S, Cell Signaling), donkey anti-guinea pig Alexa fluor 488/594 (Jackson ImmunoResearch Laboratories Inc.) and donkey anti-rabbit Alexa fluor 488/594 (ThermoFisher) – conjugated secondary antibodies.

### Urinalysis

Urine samples from P0-P1 animals were obtained by bladder puncture directly after euthanization. The QuantiChrom Creatinine Assay Kit (BioAssay Systems) was used for urine creatinine measurement. Urine albumin was measured using the Albumin Blue Fluorescent Assay Kit (Active Motif) and normalized to standard dilutions prepared from mouse serum albumin (Equitech Bio Inc.).

### Statistics

The statistical analysis was performed with GraphPad Prism 9.3.1. We used the Log-rank (Mantel-Cox) test for the Kaplan–Meier analysis. We performed nonparametric two-tailed Whitney-Mann tests for the ultrastructural and histopathological analyses.

### Supplementary Information

Supplementary Figures.

## Data Availability

All data generated or analyzed during this study are included in this published article (and its Supplementary Information files).
