# Supplementary material for: Quantifiable and reproducible phenotypic assessment of a constitutive knockout mouse model for congenital nephrotic syndrome of the Finnish type
Source: Sci Rep. 2024 Jul 10;14:15916. doi: 10.1038/s41598-024-64883-y (PMC11237045; doi:10.1038/s41598-024-64883-y)
Supplement: Supplementary file 1 — Supplementary Figures. [file 41598_2024_64883_MOESM1_ESM.pptx]

## Slide 1
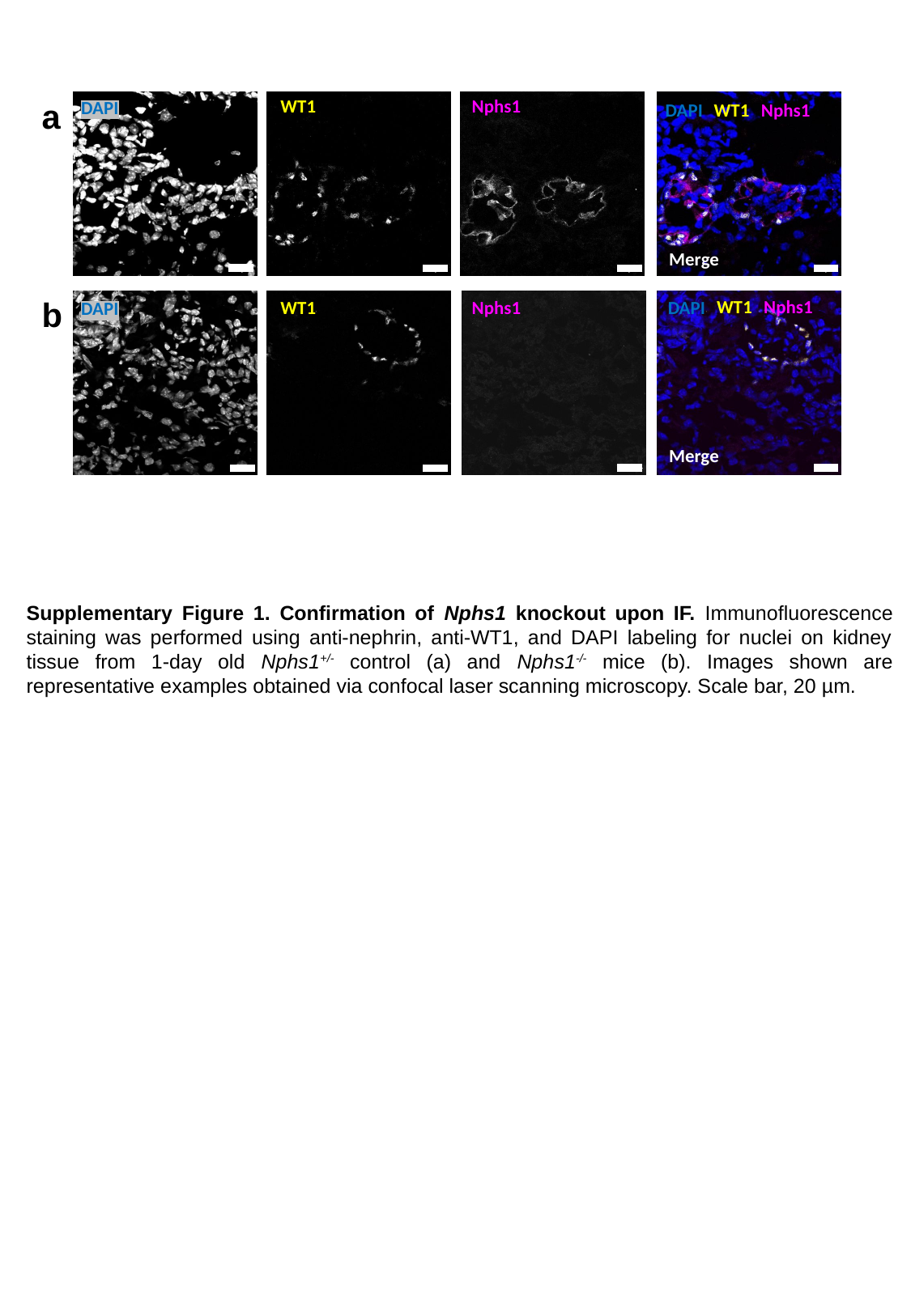

WT1
Nphs1
a
DAPI
WT1
Nphs1
DAPI
Merge
b
WT1
Nphs1
DAPI
WT1
Nphs1
DAPI
Merge
Supplementary Figure 1. Confirmation of Nphs1 knockout upon IF. Immunofluorescence staining was performed using anti-nephrin, anti-WT1, and DAPI labeling for nuclei on kidney tissue from 1-day old Nphs1+/- control (a) and Nphs1-/- mice (b). Images shown are representative examples obtained via confocal laser scanning microscopy. Scale bar, 20 µm.

## Slide 2
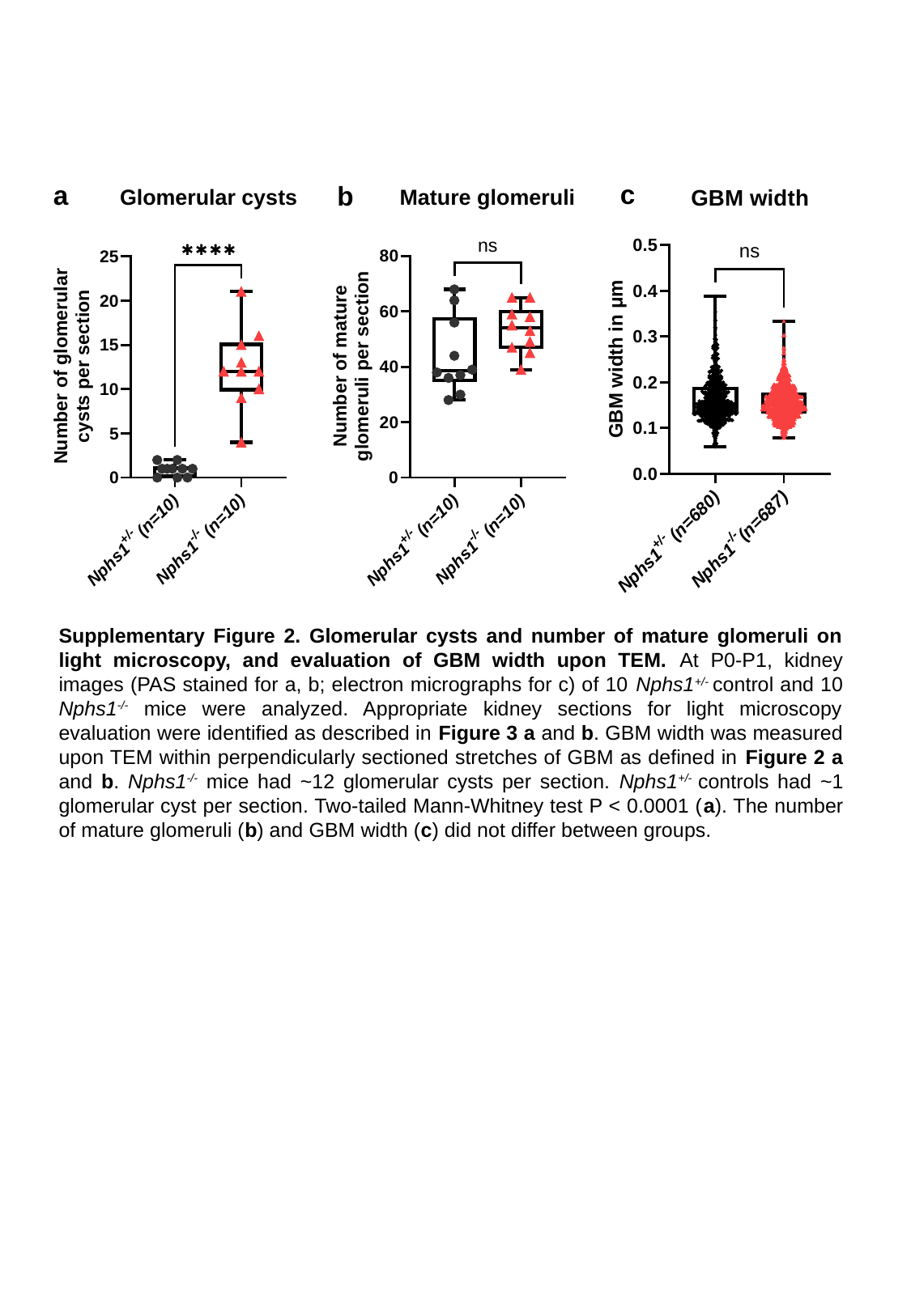

c
a
b
Supplementary Figure 2. Glomerular cysts and number of mature glomeruli on light microscopy, and evaluation of GBM width upon TEM. At P0-P1, kidney images (PAS stained for a, b; electron micrographs for c) of 10 Nphs1+/- control and 10 Nphs1-/- mice were analyzed. Appropriate kidney sections for light microscopy evaluation were identified as described in Figure 3 a and b. GBM width was measured upon TEM within perpendicularly sectioned stretches of GBM as defined in Figure 2 a and b. Nphs1-/- mice had ~12 glomerular cysts per section. Nphs1+/- controls had ~1 glomerular cyst per section. Two-tailed Mann-Whitney test P < 0.0001 (a). The number of mature glomeruli (b) and GBM width (c) did not differ between groups.

## Slide 3
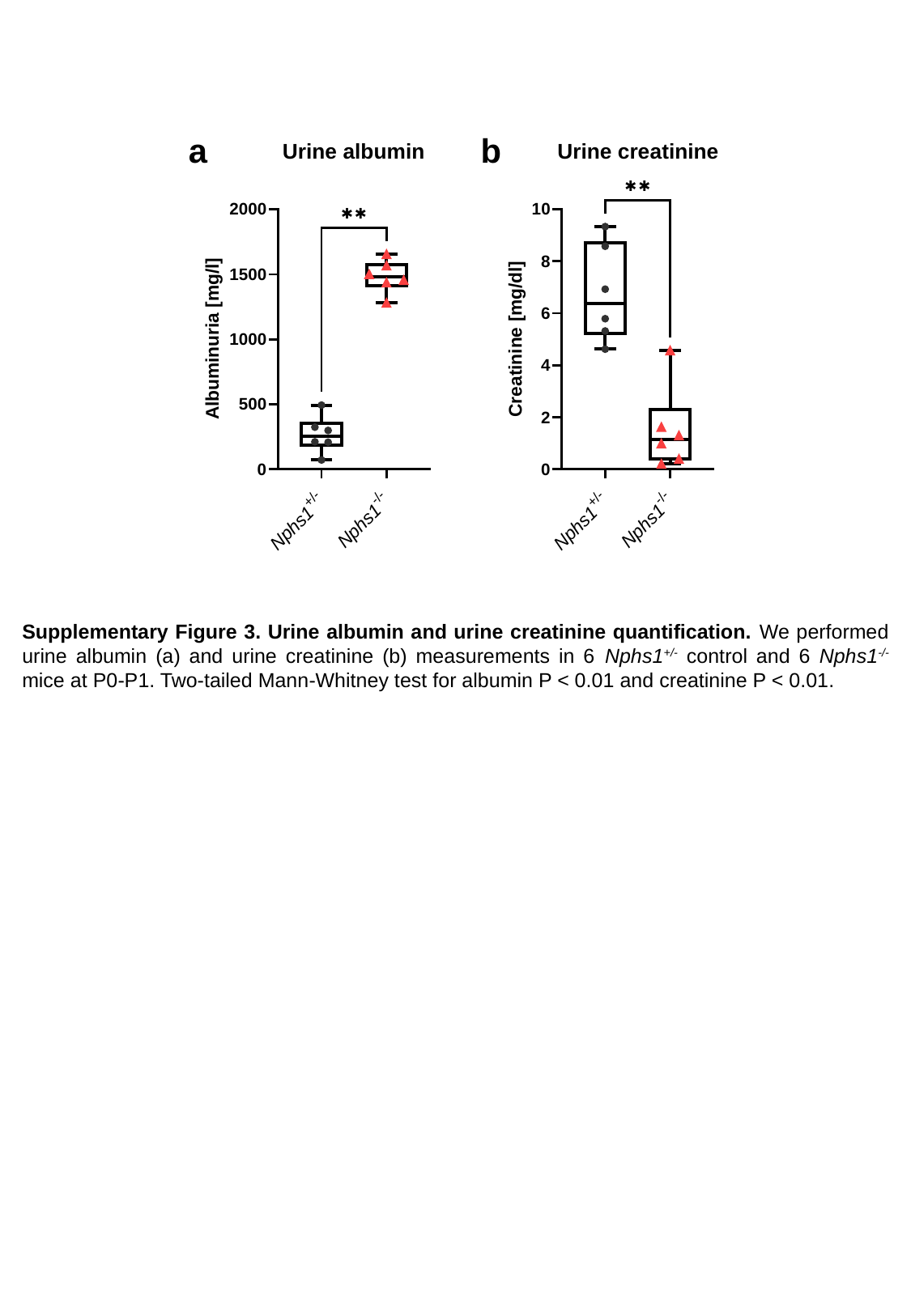

b
a
Supplementary Figure 3. Urine albumin and urine creatinine quantification. We performed urine albumin (a) and urine creatinine (b) measurements in 6 Nphs1+/- control and 6 Nphs1-/- mice at P0-P1. Two-tailed Mann-Whitney test for albumin P < 0.01 and creatinine P < 0.01.
